# Ruptured Mycotic Internal Carotid Artery Pseudoaneurysm Presenting as Massive Hematemesis in a Child

**DOI:** 10.1002/ccr3.73897

**Published:** 2026-11-10

**Authors:** Mohanned Mhairat, Mu'taz I. Alfreahat, Amjed Adnan Tarifi, Farid A. Aladham, Fatehi M. Malaysheh, Lana Alzayadneh

**Affiliations:** ^1^ Department of Pediatrics Gastroenterology The Specialty Hospital Amman Jordan; ^2^ Department of Pediatrics The Specialty Hospital Amman Jordan; ^3^ Department of Special Surgery, Faculty of Medicine Hashemite University Zarqa Jordan; ^4^ Department of Interventional Radiology The Specialty Hospital Amman Jordan; ^5^ School of Medicine The University of Jordan Amman Jordan

**Keywords:** extracranial carotid aneurysm, internal carotid artery, mycotic pseudoaneurysm, pediatric hematemesis

## Abstract

Massive hematemesis in children may rarely originate from vascular etiologies such as mycotic internal carotid artery pseudoaneurysm. Early suspicion and multidisciplinary management are crucial. Our case highlights the importance of considering vascular etiologies in unexplained bleeding and demonstrates that endovascular stenting can provide effective, life‐saving treatment.

## Graphical abstract

Contrast‐enhanced CT of the neck demonstrating a right internal carotid artery pseudoaneurysm (asterisk), surrounded by a localized fluid collection with a thin enhancing wall, consistent with a right carotid space abscess. Associated inflammatory changes obliterate the right parapharyngeal fat planes and cause mild compression of the upper airway.

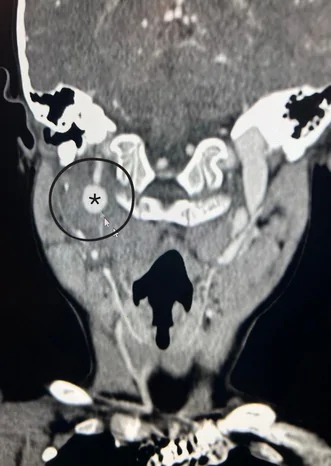

## 1 Introduction

Hematemesis in children is uncommon and is most frequently attributed to gastrointestinal causes, including erosive esophagitis, gastritis, gastroduodenal ulcers, coagulopathy, and variceal bleeding [1, 2, 3]. Extra‐gastrointestinal sources are rare but should be considered, particularly when initial evaluation is inconclusive. Cervical vascular abnormalities, including extracranial carotid artery aneurysms, represent a rare but potentially fatal cause of hematemesis in pediatric patients due to their close anatomical relationship with the pharynx and esophagus [4, 5, 6]. Among these, mycotic carotid pseudoaneurysms in children are extremely uncommon, often arising as complications of deep neck infections and carrying a high risk of rupture [7].

## 2 Case Presentation

A previously healthy, fully vaccinated 5‐year‐old boy presented to the emergency department with two episodes of large‐volume hematemesis occurring 1 h apart. Each episode consisted of fresh blood and clots, without preceding retching, vomiting, or abdominal pain. There was no history of prior epistaxis, hemoptysis, trauma, or bleeding from other sites.

Two weeks earlier, he had developed progressive right‐sided neck swelling, initially presumed to be tonsillitis or mumps, for which no treatment was received. There was no history of fever, cough, weight loss, or recent dental procedures. His past medical history and family history were unremarkable.

On arrival, his vital signs were within normal limits for age, and he was alert and hemodynamically stable. Physical examination showed a non‐pulsatile swelling over the right side of the neck, without overlying erythema, along with tender cervical lymphadenopathy. There was no pallor, respiratory distress, or neurological deficits. The abdomen was soft and non‐tender with no hepatosplenomegaly.

## 3 Methods

Laboratory results revealed a hemoglobin level of 10.8 g/dL, white blood cell count of 12 × 10^3^/μL, and C‐reactive protein of 18 mg/L. The coagulation profile, platelet count, renal function, and liver function enzymes were all within normal limits. Abdominal ultrasound findings were unremarkable. Given the presentation with hematemesis, gastrointestinal causes were initially considered. However, the absence of preceding vomiting or retching, epistaxis, or hemoptysis, together with the recent progressive unilateral neck swelling, also raised concern for an upper aerodigestive or cervical source of bleeding.

Upper gastrointestinal endoscopy performed 4 h after presentation excluded a gastric or esophageal source but revealed active bleeding from the oropharynx, prompting further evaluation for a deep neck process and an associated vascular complication. Two hours later, he developed another episode of hematemesis with a rapid drop in hemoglobin to 6.6 g/dL, prompting transfer to the intensive care unit and transfusion of one unit of packed red blood cells.

Contrast‐enhanced computed tomography (CECT) of the neck demonstrated a 1 cm saccular mycotic pseudoaneurysm of the right internal carotid artery, located approximately 3.5 cm distal to the bifurcation, associated with a 4.2 × 1.8 cm parapharyngeal abscess causing airway narrowing (Figures 1 and 2). CECT demonstrated fluid and inflammatory changes in the right carotid space extending to the wall of the internal carotid artery pseudoaneurysm, with adjacent arterial‐wall thickening. These findings are supported by contiguous inflammatory involvement of the artery by the parapharyngeal space. No carotid‐pharyngeal fistula was identified on imaging or during surgical assessment, and the pharyngeal and esophageal walls appeared intact. Although the pseudoaneurysm was considered the source of the oropharyngeal hemorrhage based on the endoscopic and anatomical findings, the exact route by which bleeding entered the pharynx could not be established.

**FIGURE 1:**
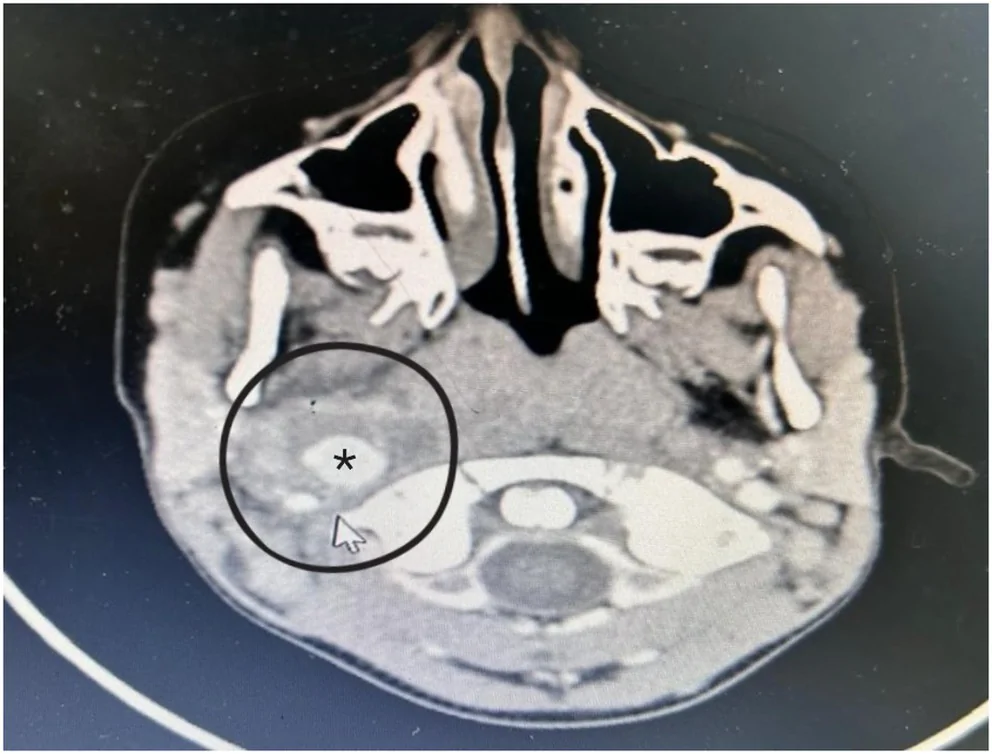
Axial contrast‐enhanced computed tomography (CT) of the neck demonstrates a well‐defined saccular pseudoaneurysm arising from the right internal carotid artery (star), surrounded by ill‐defined inflammatory soft‐tissue changes within the right carotid space.

**FIGURE 2:**
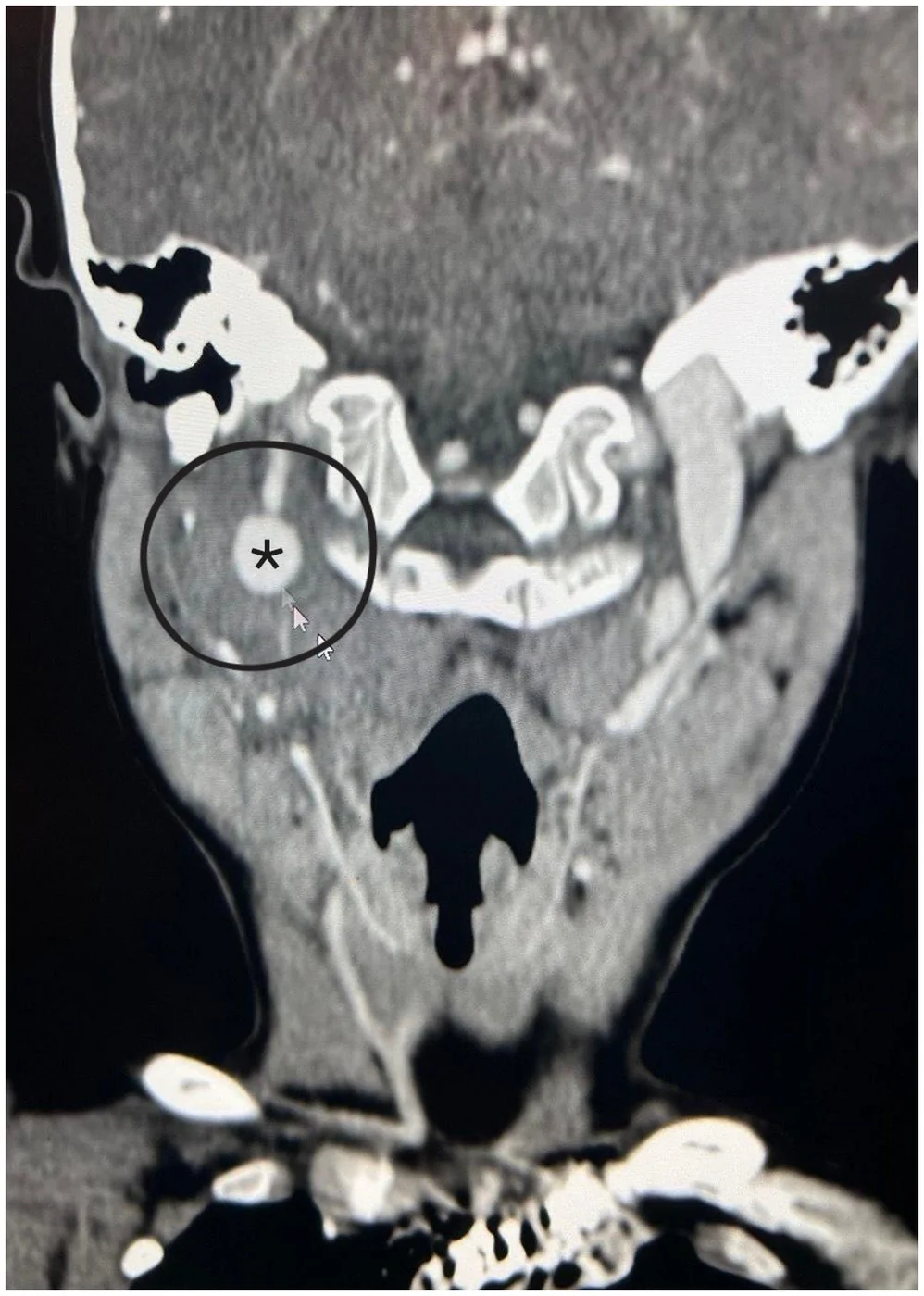
Coronal contrast‐enhanced CT of the neck demonstrating a right internal carotid artery pseudoaneurysm (star) surrounded by a localized fluid collection with a thin enhancing wall consistent with a right carotid space abscess. The inflammatory changes completely obliterate the right parapharyngeal fat planes and mildly compress the upper airway.

A multidisciplinary team involving interventional radiology, otolaryngology, infectious disease, and neurosurgery was urgently convened. Treatment options, including parent‐vessel sacrifice and reconstructive endovascular treatment, were discussed with the patient's family. The family declined parent‐vessel sacrifice because of the associated risks, and covered‐stent reconstruction was selected to exclude the pseudoaneurysm while preserving right internal carotid artery flow. The patient was loaded with aspirin and clopidogrel before the procedure. Cerebral angiography was performed under general anesthesia through right common femoral arterial access. Intravenous heparin was administered during the procedure to maintain an activated clotting time of approximately 200 s. A 6 × 37 mm Lifestream covered stent (BD) was successfully deployed across the pseudoaneurysm, achieving complete exclusion of the lesion with preserved right internal carotid artery flow. Surgical drainage and packing of the parapharyngeal abscess were subsequently performed (Figures 3 and 4).

**FIGURE 3:**
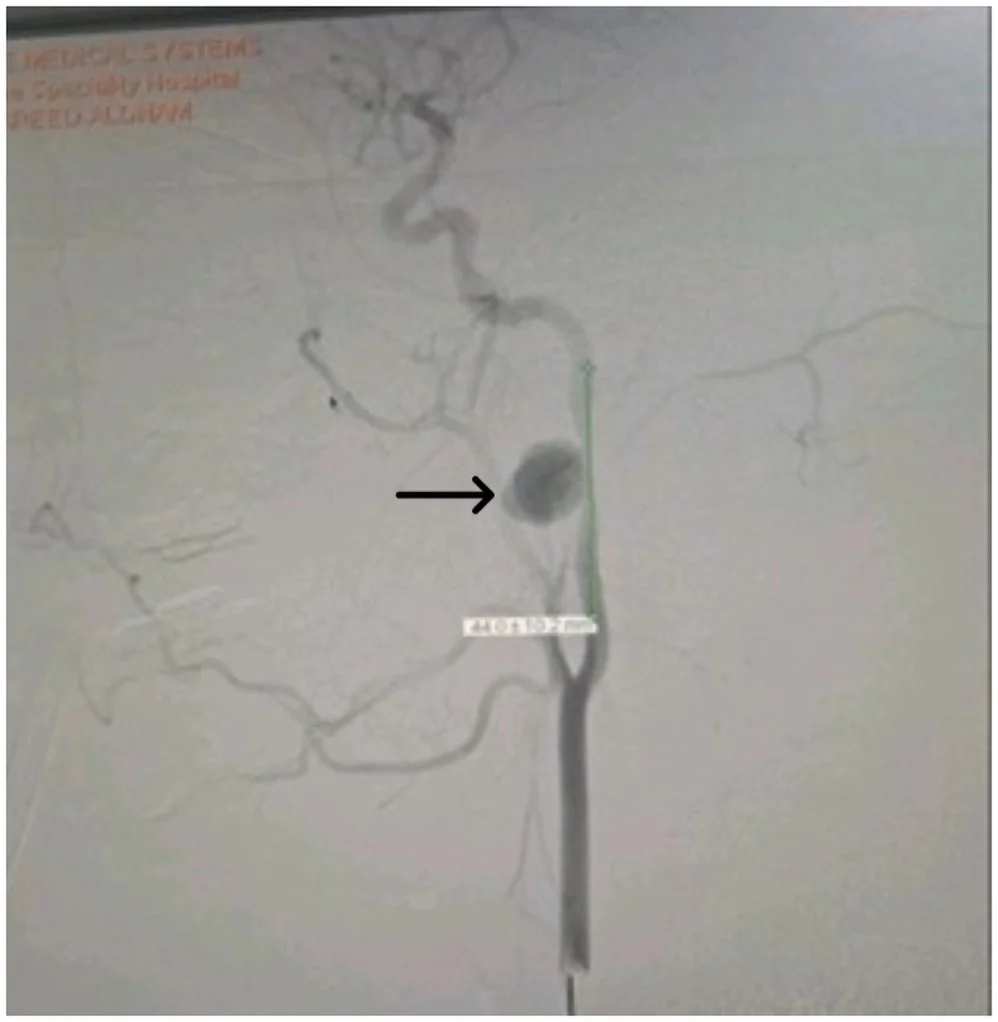
Digital subtraction angiography showing a saccular pseudoaneurysm (arrow) of the distal cervical right internal carotid artery, with features suggestive of an infectious etiology.

**FIGURE 4:**
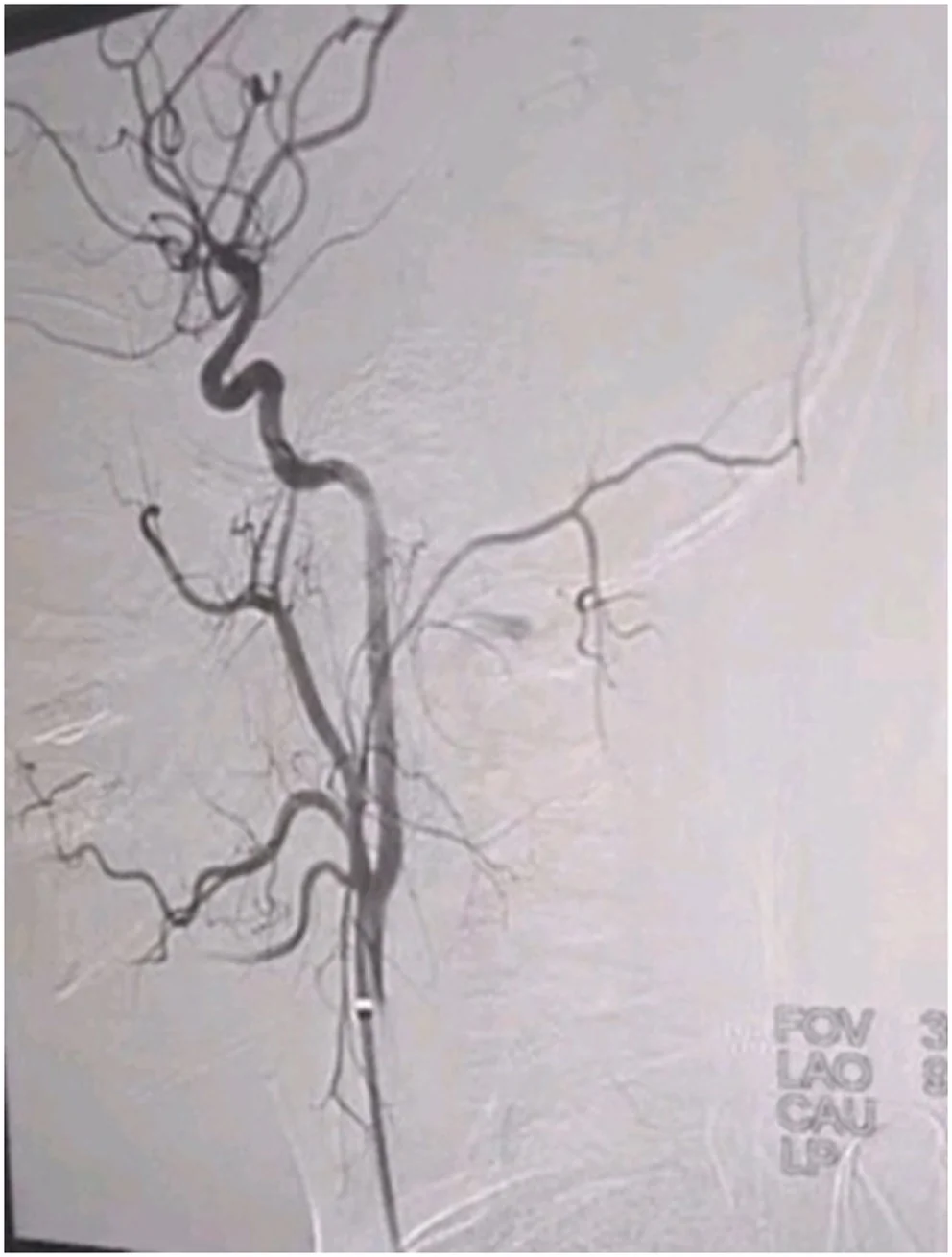
Post‐intervention digital subtraction angiography showing complete exclusion of the pseudoaneurysm following covered stent deployment, with preserved right internal carotid artery flow.

Broad‐spectrum intravenous antibiotics (ceftriaxone, vancomycin, and metronidazole) were initiated to provide aerobic and anaerobic coverage. Blood cultures remained sterile, and echocardiography excluded infective endocarditis. No specimen from the parapharyngeal collection was sent for microbiological culture or histopathological examination; therefore, microbiological and histopathological confirmation of the infectious etiology was not available. Nevertheless, the close anatomical relationship between the pseudoaneurysm and the adjacent parapharyngeal abscess, together with the clinical and radiological response to prolonged antimicrobial therapy, supported an infectious etiology.

## 4 Conclusion and Results

The patient was extubated on the second postoperative day, with cessation of bleeding and progressive clinical improvement. Hemoglobin stabilized at 12.9 g/dL by Day six. Following stent placement, dual antiplatelet therapy was continued with aspirin 100 mg once daily and clopidogrel 75 mg once daily for 1 week, followed by clopidogrel 37.5 mg once daily. Platelet function testing at 2 weeks showed a P2Y12 reaction unit (PRU) value of 152. Follow‐up magnetic resonance imaging (MRI) at 6 weeks showed near‐complete resolution of the abscess and thrombosis of the pseudoaneurysm. He completed a six‐week course of intravenous antibiotics. He was discharged in stable condition with a cervical collar and instructions for repeat imaging. Longer‐term cerebral and neck CT angiography demonstrated patency of the stented cervical segment of the right internal carotid artery, with normal vessel diameter and opacification and no recurrent aneurysmal dilatation (Figure 5). At 12‐month follow‐up, the patient remained asymptomatic without recurrent hematemesis, neck swelling, neurological deficits, or evidence of infection recurrence. He remained on aspirin 100 mg and clopidogrel 37.5 mg once daily, planned to continue until 18 months after stent placement followed by reassessment. No additional vascular intervention was required.

**FIGURE 5:**
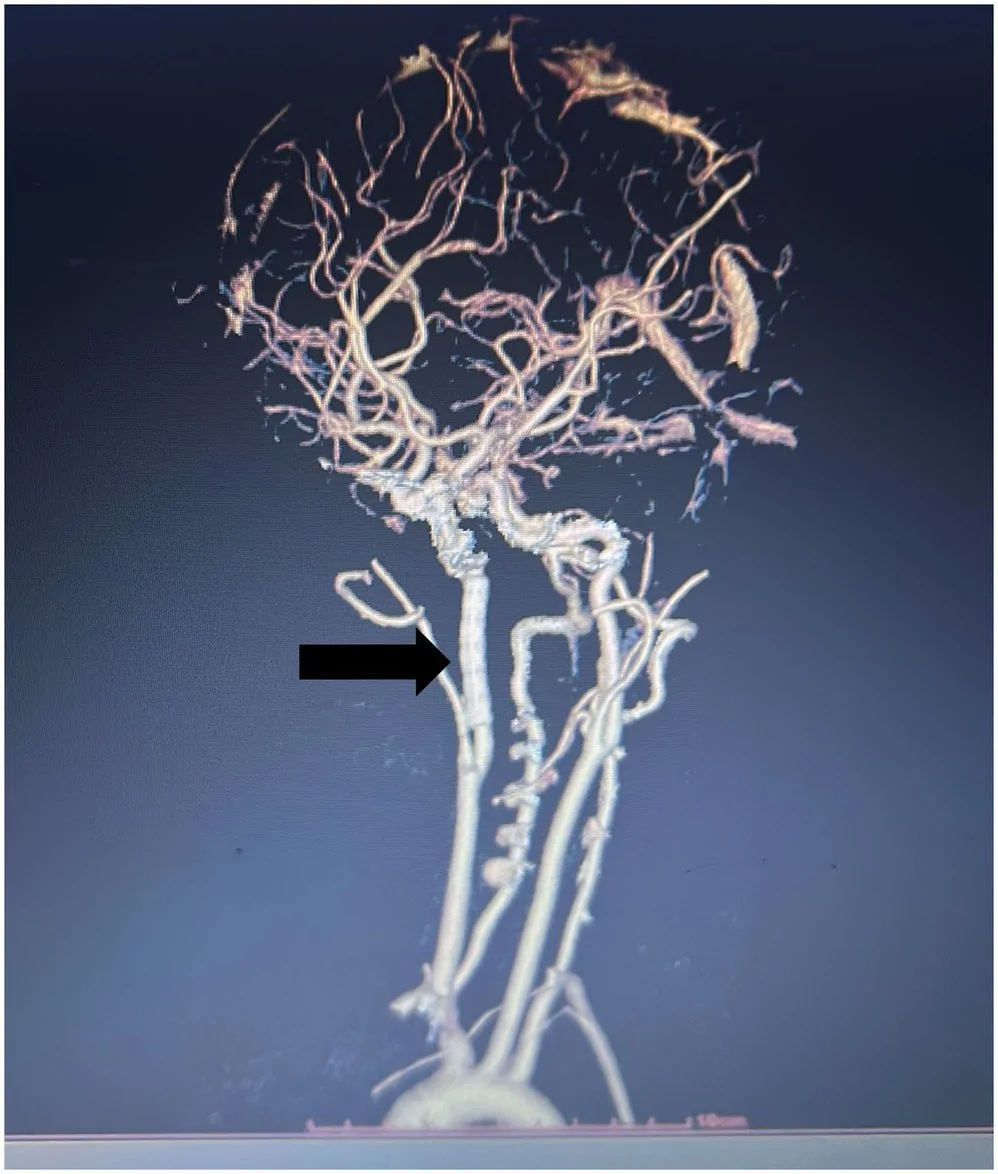
Follow‐up cerebral and neck CT angiography with three‐dimensional volume‐rendered reconstruction demonstrating patency of the stented cervical segment of the right internal carotid artery (arrow) with preserved arterial opacification.

## 5 Discussion

Mycotic aneurysms of the extracranial carotid artery are exceedingly rare in the pediatric population [8]. Less than 50 cases are reported worldwide in the age distribution reported in Hanger and Baker's systematic review of infective native extracranial carotid artery pseudoaneurysm [9]. In pediatrics, these aneurysms predominantly occur as a result of infection or trauma [8, 10], rather than the atherosclerotic or degenerative processes observed in adults [11]. Mycotic aneurysms are pathologically infectious pseudoaneurysms, arising from localized destruction of the arterial wall, allowing blood to extravasate into surrounding tissues and form a sac that remains in continuity with the arterial lumen [12].

These lesions usually arise from direct spread from adjacent deep neck infection, such as parapharyngeal or retropharyngeal abscesses, or septic embolization, both of which weaken the arterial wall and increase its susceptibility to rupture [8, 13, 14, 15]. In mycotic pseudoaneurysms, the cervical portion of the internal carotid artery is particularly vulnerable in pediatric patients due to its close anatomical relationship with regional lymphatic chains and deep neck spaces [7, 16]. Children with extracranial carotid pseudoaneurysms clinically present with a pulsatile cervical mass, neck pain, fever, dyspnea, and dysphagia [6, 17, 18, 19, 20]. Although microbiological confirmation was unavailable, the clinical and radiological findings strongly supported an infectious etiology. Alternative causes included traumatic or dissection‐related pseudoaneurysm and an underlying congenital or connective tissue abnormality. A traumatic origin was less likely given the absence of a history of trauma, while CECT and CT angiography showed no evidence of dissection. Congenital or connective tissue causes were not formally excluded but were less favored given the preceding neck symptoms and adjacent parapharyngeal abscess. Presentation with hematemesis is exceptionally rare and generally occurs when the pseudoaneurysm erodes into the pharynx or upper aerodigestive tract [5, 6, 21]. In our patient, upper gastrointestinal endoscopy excluded a gastric or esophageal source and demonstrated active oropharyngeal bleeding, while vascular imaging identified the adjacent internal carotid artery pseudoaneurysm. This highlights the need for considering broader differential diagnoses and avoiding diagnostic anchoring when evaluating pediatric hematemesis, especially in patients with recent or unresolved neck infection [22, 23].

Although no discrete carotid‐pharyngeal fistula was demonstrated and the pharyngeal wall appeared intact at subsequent assessment, a transient communication or focal erosion present during the acute bleeding episode could have allowed blood to enter the pharynx and subsequently be swallowed and vomited. However, this mechanism could not be confirmed, and the exact route by which the bleeding entered the pharynx remains uncertain.

Diagnosis of carotid artery pseudoaneurysm in children remains challenging [24]; therefore, imaging plays a central role in diagnosis. While catheter angiography is the gold standard for mycotic pseudoaneurysm diagnosis, CECT and MRI provide high diagnostic accuracy and are often sufficient for initial evaluation [25], allowing for rapid diagnosis of life‐threatening vascular lesions. Early diagnosis is critical, as mycotic pseudoaneurysms carry a high risk of serious complications [26], including rupture, stroke, bleeding, and airway obstruction [27]. Mortality rates have been reported to reach up to 77% in untreated cases and remain high even with intervention [28]. Although most reported mycotic aneurysms involve larger vessels such as the aorta [29], similar principles of diagnosis and management apply across vascular territories, emphasizing the need for early imaging, antimicrobial treatment, and early intervention [30]. Management of pediatric mycotic aneurysm is complex and must be individualized [31]. Surgical ligation of the internal carotid artery carries a high risk, especially when the lesion is located close to the skull base, where proximal vascular control is limited, and is associated with significant morbidity and mortality [31, 32]. Endovascular approaches, including covered stent placement or embolization [33, 34], have therefore emerged as a preferred option in selected pediatric patients, offering effective exclusion of the lesion while preserving cerebral perfusion [35, 36]. The utilization of stents or embolization in pediatric patients is still limited due to insufficient long‐term outcome data and ambiguity regarding the ideal antiplatelet therapy, necessitating meticulous patient selection, multidisciplinary decision‐making, and continuous follow‐up [13, 37].

This case contributes to the limited pediatric literature by illustrating an unusually severe and rapidly progressive presentation, with a rapid decline in hemoglobin levels and a life‐threatening hemorrhage occurring within hours of admission. Successful endovascular stenting, combined with aggressive antimicrobial therapy and surgical drainage of the abscess, resulted in a favorable outcome without neurological sequelae, with no recurrence observed during 12 months of follow‐up.

Infectious pseudoaneurysm of the extracranial internal carotid artery is a rare but life‐threatening complication of deep neck infection. It may present with atypical and misleading manifestations, including hematemesis, which can delay diagnosis and increase the risk of catastrophic hemorrhage. This case serves as a reminder for clinicians to consider non‐gastrointestinal causes when assessing unexplained upper aerodigestive tract bleeding in pediatric patients with a recent neck infection. Prompt recognition, appropriate imaging, and early multidisciplinary management are crucial and can be lifesaving.

## Author Contributions

**Mohanned Mhairat:** conceptualization, investigation, resources, writing – review and editing. **Mu'taz I. Alfreahat:** data curation, investigation, resources, writing – review and editing. **Amjed Adnan Tarifi:** data curation, investigation, visualization, writing – review and editing. **Farid A. Aladham:** investigation, resources, visualization, writing – review and editing. **Fatehi M. Malaysheh:** data curation, investigation, resources, writing – review and editing. **Lana Alzayadneh:** conceptualization, data curation, resources, supervision, writing – original draft, writing – review and editing.

## Funding

The authors have nothing to report.

## Consent

Written informed consent for publication was obtained from the patient's parent in accordance with journal guidelines, and a copy is provided in the supplementary files.

## Data Availability

Availability of supporting data: All relevant data supporting the findings of this case are included within the article and its supplementary material.
